# Effect of weight gain on blood pressure in Ugandan persons with HIV on dolutegravir/lamivudine/ tenofovir disoproxil fumarate over 48 weeks

**DOI:** 10.1371/journal.pone.0325020

**Published:** 2025-06-11

**Authors:** Willington Amutuhaire, Barbara Castelnuovo, Nele Brusselaers, Martin Nabwana, Lal Muhammad, Brendan Maloney, Bridgette Nixon, Jean-Marc Schwarz, Frank Mulindwa

**Affiliations:** 1 Yale School of Medicine, Department of Cardiology, Connecticut, United States of America; 2 Infectious Diseases Institute, Makerere University Kampala, Uganda; 3 Global Health Institute, Antwerp University, Antwerp, Belgium; 4 Department of Women’s and Children’s Health, Tumour and Cell Biology, Karolinska University, Sweden; 5 Makerere University Johns Hopkins Collaboration, HIV clinic, Kampala, Uganda; 6 United Health Services, Wilson Hospital, Department of Internal Medicine, New York, United States of America; 7 SUNY Upstate Medical University, Medical School, New York, United States of America; 8 University of California San Francisco, School of Medicine, San Francisco, United States of America; 9 Touro University California College of Osteopathic Medicine, Department of Basic Sciences, Vallejo, California, United States of America; Makerere University, UGANDA

## Abstract

**Background:**

Most people living with HIV in low and middle-income countries are taking fixed dose combination tenofovir disoproxil fumarate/lamivudine/dolutegravir (TLD). Dolutegravir use has been associated with weight gain, a known risk factor for hypertension. We aimed to determine if weight gain in Ugandan anti-retroviral therapy (ART) naïve patients on TLD correlated with increase in blood pressure.

**Methods:**

We analyzed data from the ‘Glucose metabolism changes in Ugandan persons with HIV (PLHIV) on Dolutegravir (GLUMED)’ study which was a prospective cohort study with ART naïve persons with HIV ≥ 18 years followed up on TLD over 48 weeks. A scatter plot with 95% confidence intervals and regression line illustrating the relationship between weight change and mean arterial pressure (MAP) change from baseline to 48 weeks was created. To further examine the effect of weight change on MAP, we performed a linear regression analysis, with MAP change as the dependent variable and weight change as the independent variable.

**Results:**

Of the 220 patients’ data analyzed, 129 (58.6%) were female, the median baseline age was 31 years (interquartile range (IQR): 27.0–38.0), the median baseline CD4 cell count was 319 cells/mm^3^ (IQR 160.0–524.0). The median weight gain over 48 weeks was 3.0 (IQR: −0.1–6.3). We found a moderate positive linear relationship between weight gain and MAP over 48 weeks. For every increase in weight of 1 kg over 48 weeks, there was an adjusted increase in MAP by 0.62mmHG.

**Conclusion:**

We provide additional evidence to suggest that the noticed weight gain after starting dolutegravir based ART may be associated with a heightened risk of incident hypertension.

## Background

Over 80% persons living with HIV in low and middle-income countries were taking fixed dose combination tenofovir disoproxil fumarate/lamivudine/dolutegravir (TLD) by mid-2024, more so countries supported by the U.S. President’s Emergency Plan for AIDS Relief [1,2]. The widespread uptake followed WHO recommendations in 2016 and later 2018 recommending dolutegravir (DTG) anchored anti-retroviral therapy (ART) use as first line and optional second line therapy, respectively [3,4]. The recommendations followed reports of increased resistance to non-nucleoside reverse transcriptase inhibitors (NNRTIs) as well as multiple advantages of DTG including; having a high genetic barrier to resistance, a good side effect profile, high efficacy and less drug-drug interactions [5,6]. Dolutegravir use however has been associated with weight gain when compared to other anchor drugs like non-nucleoside reverse transcriptase inhibitors and protease inhibitors [7–10].

Obesity is a risk factor for dyslipidemia, glucose dysmetabolism, cardiovascular disease and hypertension [11–13]. With mortality and morbidity in persons living with HIV (PLHIV) shifting from AIDS associated opportunistic infections to non- infectious diseases like cardiovascular diseases, renal disease, liver failure and non–AIDS defining malignancies, it is pertinent to understand the risk posed by different ART regimens to inform surveillance decisions as well as ART choice decisions in primary care [14–18]. Whether the weight gain from integrase inhibitors, in particular DTG translates into dyslipidemia, glucose dysmetabolism, cardiovascular disease and hypertension is still subject to research.

There is growing evidence to demonstrate that weight gain noticed in patients on dolutegravir is associated with increased blood pressure as compared to patients on other regimens as demonstrated by two studies with both ART naïve and experienced adults [8,19,20] We sought to evaluate in a Ugandan cohort of PLHIV if weight gain over 48 weeks on TLD correlated with changes in blood pressure. Our cohort was of particular interest because it was composed of younger participants with minimal other risk factors for hypertension.

## Methods

### Study design and setting

The participants of the study were recruited in the ‘Glucose metabolism changes in Ugandan persons living with HIV (PLHIV) on Dolutegravir (GLUMED)’ study which was a prospective cohort study at the Kisenyi Health Center IV HIV clinic, in Uganda’s capital city, Kampala [21–23]. The primary objective of the study was to describe glucose metabolism changes in participants over 48 weeks on dolutegravir based ART.

### Study participants and study processes

The GLUMED study was conducted between January/2021 and September/2023. ART naïve PLHIV aged 18 years or more were recruited excluding pregnant women and very sick patients unable to undergo a 2 hour- oral glucose tolerance test (OGTT).

Baseline demographic, clinical and social data were collected. Enrolled patients were prospectively followed up with 2 hour- oral glucose tolerance tests (OGTT), body mass index, waist circumference, blood pressure measurements, adherence counselling and assessment as well as assessment of concurrent medications at 12, 24, 36 and 48 weeks. At every visit, a protocol-based procedure was used to measure blood pressure. After a 5 minutes rest, using a Welch Allyn Vital Signs Monitor, 300 Series 53NT0®, we measured patient blood pressures with patients seated upright with their backs to the chairs, feet flat on the floor and arms supported at heart level. A full description of the study protocol is described in our earlier publications [21,24]

### Outcome

The primary outcome of the study was the change in mean arterial pressure (MAP) from baseline to 48 weeks. MAP is an index of systolic and diastolic blood pressure calculated by the formular; 1/3 systolic blood pressure + 2/3 diastolic blood pressure.

### Statistical analysis

This study aimed to assess the effect of weight change on MAP changes between baseline and 48 weeks among participants, with adjustment for relevant covariates (sex, age, physical activity, and baseline HIV clinical stage). We performed secondary analysis of patient data collected in the GLUMED study excluding patients who had hypertension and were on treatment at baseline. We also excluded patients who were diagnosed with hypertension and started on anti-hypertensives during follow up.

Descriptive statistics were used to summarize baseline demographic and clinical characteristics. Continuous variables were reported as median (interquartile range (IQR)) due to their skewed distribution, while categorical variables were reported as counts and percentages.

A scatter plot with a 95% confidence interval and regression line illustrating the relationship between weight change and MAP change was created. This plot provides a visual representation of the strength and direction of the association. To further examine this association, we performed a linear regression analysis, with MAP change as the dependent variable and weight change as the independent variable. Both unadjusted and adjusted β-coefficients with 95% confidence intervals were estimated. The adjusted model included covariates selected based on clinical relevance and potential confounding, including sex, age, physical activity, and baseline HIV clinical stage. All analyses were conducted using Stata software (version 17.0), with a two-tailed significance level set at p < 0.05 for all tests.

## Results

Of the 243 patients that completed 48 weeks of follow up in the GLUMED study, 23 patients were diagnosed with hypertension and were on hypertension treatment hence were not included in the final analysis, leaving 220 patients.

Of the 220 patients’ data analyzed, 129 (58.6%) were female. The median age of the participants was 31 years (IQR: 27.0–38.0) with a median CD4 cell count of 319.0 cells/mm^3^ (IQR: 160.0–524.0). The majority (212 (96.4%)) of the patients were in HIV clinical stage 1 with 3 (1.4%), 4 (1.8%), 1 (0.5%) being in HIV clinical stages 2,3 and 4 respectively. Seven (3.2%) patients had an established diagnosis of active tuberculosis diagnosed at enrollment into HIV care. Overall, 138 (62.7%) had a normal BMI, 48 (21.8%) were overweight, 22 (10.0%) underweight and 12 (5.5%) obese. About half (126 (57.3)) reported not taking alcohol. Most (180 (81.8%) of the patients self-reported as meeting WHO recommendations on physical activity for health. The median serum creatine, fasting LDL, fasting HDL and fasting total cholesterol at baseline were: 0.8 mg/dl, 77 mg/dl, 31.7 mg/dl and 134 mg/dl respectively. At 24 weeks of follow up, 215 (99.5%) patients had viral suppression. (baseline characteristics are summarized in Table 1)

**Table 1:** Baseline clinical and demographic characteristics of the study population.

Characteristic	N = 220
Age years, Median (IQR)	31.0 (27.0-38.0)
Baseline CD4 cell count (cells/mm^3^) Median (IQR)	319.0 (160.0-524.0)
Sex	
Female	129 (58.6)
Male	91 (41.4)
Level of education	
Uneducated	3 (1.4)
Primary	117 (53.2)
Secondary	91 (41.4)
Tertiary	9 (4.1)
Religion	
Christian	168 (76.4)
Muslim	52 (23.6)
Residence	
Rural	15 (6.8)
Urban	205 (93.2)
Employment	
No	31 (14.1)
Yes	189 (85.9)
Marital status	
Single	121 (55.0)
Married	99 (45.0)
Tuberculosis status	
No symptoms	185 (84.1)
TB suspect	28 (12.7)
TB disease	7 (3.2)
Baseline blood pressure	
Normal BP	173 (78.6)
Pre-hypertension	47 (21.4)
HIV clinical stage	
Stage 1	212 (96.4)
Stage 2	3 (1.4)
Stage 3	4 (1.8)
Stage 4	1 (0.5)
Baseline weight (kg)	60.0 (52.5-66.5)
Body Mass Index (BMI)	
Underweight (<18.5)	22 (10.0)
Normal (18.5–24.9)	138 (62.7)
Overweight (25.0–29.9)	48 (21.8)
Obese (≥ 30]	12 (5.5)
Waist circumference	
Normal	149 (67.7)
Increased risk of cardiometabolic complications	37 (16.8)
Substantially increased risk of cardiometabolic	34 (15.5)
Smoking status	
Smoker	14 (6.4)
Non-smoker	206 (93.6)
Physical activity	
GPAQ<600 MET minutes	40 (18.2)
GPAQ≥600 MET minutes	180 (81.8)
Alcohol consumption	
No consumption	126 (57.3)
Low risk alcohol consumption	58 (26.4)
Hazardous alcohol consumption	19 (8.6)
Risk of alcohol dependence	17 (7.7)
24-week viral loads (Proxy baseline VL)	
Virologically suppressed	215 (99.5)
Unsuppressed Viral load	1 (0.5)
Net weight change after 48 weeks	3.0 (−0.1-6.3)
Change in BMI from baseline to Week 48	1.1 (0.0-2.5)
Laboratory investigations, Median (IQR)	
Baseline creatinine(mg/dl)	0.8 (0.7-0.9)
Baseline LDL(mg/dl)	77.0 (58.2-93.0)
Baseline HDL(mg/dl)	31.7 (25.5-39.8)
Baseline Total cholesterol(mg/dl)	134.0 (117.8-157.8)
Baseline Triglycerides(mg/dl)	90.3 (68.6-116.9)

*Data are presented as median (IQR) for continuous measures, and n (%) for categorical measures. LDL- Low density lipoproteins, HDL- High density lipoprotein, VL- Viral load, GPAQ- Global Physical Activity Questionnaire, TB- Tuberculosis. BMI, HIV clinical stage, physical activity and waist circumference were categorized according to the WHO cutoffs* [25–28]*. Blood pressure was categorized according to the Joint National Committee 8 (JNC-8) guidelines* [29]*. Virologic suppression was categorized according to the Uganda HIV treatment guidelines* [30].

### Effect of change in weight on change in mean arterial pressure

**Fig 1:**
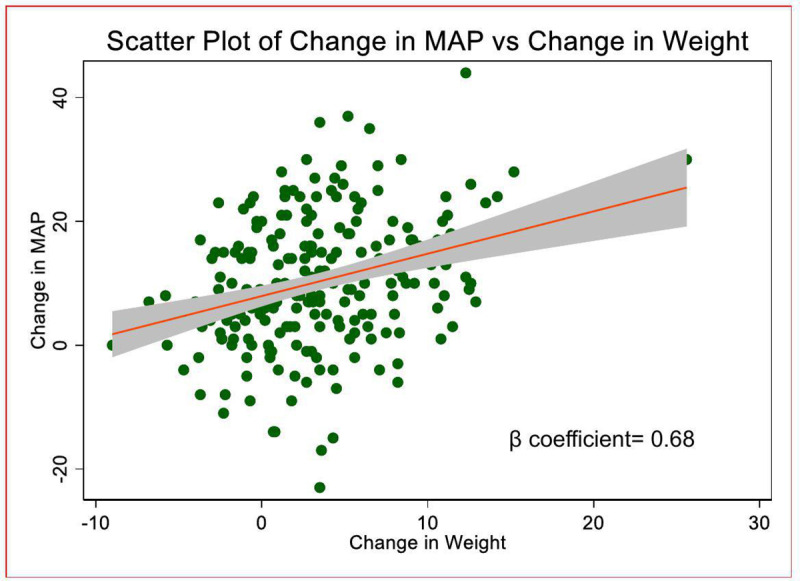
Scatter plot of change in mean arterial pressure versus change in weight with 95% confidence intervals.

We found a moderate positive linear relationship between weight gain and MAP over 48 weeks. On linear regression to determine the effect of change in weight over 48 weeks on change in MAP, after adjustment for sex, age, physical activity, and baseline HIV clinical stage in the final model, we found a significant adjusted β co-efficient of 0.62 (0.37, 0.86) (P < 0.001). The observed change in MAP was independent of other factors known to be risk factors for hypertension including baseline BMI, baseline renal function and waist circumference [31–33]. Table 2, Fig 1.

**Table 2:** Linear regression evaluating the effect of net weight change from baseline to 48 weeks on change in mean arterial pressure.

Characteristic	Unadjusted β Coefficient (95% CI)	P-value	Adjusted β Coefficient (95% CI)	P-value
Net weight change after 48 weeks	0.68 (0.46, 0.91)	**<0.001**	0.62 (0.37, 0.86)	**<0.001**
Age (years)	0.17 (−0.04, 0.37)	0.106	0.07 (−0.14, 0.27)	0.520
Baseline CD4 cell count (cells/mm^3^)	0 (−0.01, 0.00)	0.253		
Sex				
Female	Ref			
Male	0.76 (−2.13, 3.66)	0.604	0.26 (−2.57, 3.10)	0.854
Residence				
Rural	Ref			
Urban	−0.89 (−6.93, 5.14)	0.770		
Employment				
No	Ref			
Yes	0.05 (−3.90, 3.99)	0.981		
Marital status				
Single	Ref			
Married	2.93 (0.10, 5.75)	**0.042**		
Tuberculosis status				
No symptoms	Ref			
TB suspect	5.34 (1.27, 9.40)	**0.010**		
TB disease	−1.38 (−9.28, 6.52)	0.731		
Baseline blood pressure				
Normal BP	Ref			
Pre-hypertension	−7.31 (−10.74,-3.88)	**<0.001**		
HIV clinical stage				
Stage 1	Ref			
Stage 2	8.06 (5.19,10.93)	**<0.001**	4.34 (−2.48,11.17)	0.211
Stage 3	13.56 (5.42,21.69)	**0.001**	11.27 (2.46,20.08)	**0.012**
Stage 4	0.06 (−1.39, 1.50)	0.939	−3.01 (−5.36,-0.66)	**0.012**
Body Mass Index (BMI)				
Underweight (<18.5)	Ref			
Normal (18.5–24.9)	0.69 (−4.62, 5.99)	0.799		
Overweight (25.0–29.9)	−0.93 (−6.60, 4.74)	0.747		
Obese (≥ 30]	3.01 (−3.61, 9.63)	0.372		
Waist circumference				
Normal	Ref			
Increased risk of cardiometabolic complications	0.35 (−3.83, 4.53)	0.868		
Substantially increased risk of cardiometabolic	1.21 (−2.23, 4.66)	0.489		
Smoking status				
Non-Smoker	Ref			
Smoker	−1.62 (−8.96, 5.72)	0.665		
Physical activity				
GPAQ<600 MET minutes	Ref			
GPAQ≥600 MET minutes	0.28 (−3.10, 3.65)	0.872	−0.22 (−3.50, 3.06)	0.895
Alcohol consumption				
No consumption	Ref			
Low risk alcohol consumption	−1.4 (−4.46, 1.65)	0.366		
Hazardous alcohol consumption	4.73 (0.19, 9.26)	**0.041**		
Risk of alcohol dependence	4.62 (−2.71,11.96)	0.215		
Change in BMI from baseline to Week 48	1.8 (1.22, 2.39)	**<0.001**		
Laboratory investigations				
Baseline creatinine(mg/dl)	4.82 (−0.06, 9.70)	0.053		
Baseline LDL (mg/dl)	0 (−0.06, 0.06)	0.997		
Baseline HDL (mg/dl)	−0.13 (−0.29, 0.02)	0.095		
Baseline Total cholesterol(mg/dl)	0.01 (−0.04, 0.05)	0.795		
Baseline Triglycerides(mg/dl)	0.03 (−0.00, 0.07)	0.075		

*MET- Metabolic Equivalent of Task, LDL- Low density lipoproteins, HDL- High density lipoproteins,* GPAQ- Global Physical Activity Questionnaire*. The adjusted model included the following covariates: sex, age, physical activity, and baseline HIV clinical stage*

**Fig 1** On linear regression, after adjustment for sex, age, physical activity, andbaseline HIV clinical stage in the final model, the adjusted β co-efficient was 0.62 (0.37, 0.86) (P < 0.001).

## Discussion

In this study, we determined the effect of weight gain on blood pressure (expressed as mean arterial pressure) over 48 weeks on TLD. We found a moderate positive linear relationship between weight gain and MAP with an increase of 0.68mmHG for every 1 kg increase in body weight over 48 weeks with consistent findings after adjusting for baseline age, sex, physical activity and baseline clinical stage.

Currently, the most used ART is fixed dose combination TLD in more than 80% PLHIV in low-middle income countries [1,2]. Dolutegravir based ART has been shown to induce weight gain compared to other anchor drugs [7–10]. Increased adiposity one of the characteristics of obesity is known to cause hypertension via various complex interlinked mechanisms including sympathetic nervous system overactivation, stimulation of the renin-angiotensin-aldosterone system, alterations in adipose-derived cytokines, insulin resistance, and structural and functional renal changes [11–13]. Determining if the reported weight gain in patients on TLD correlates with increasing blood pressure can help guide physician choices when individualizing anti-retroviral therapy as well as HIV national programs in designing hypertension surveillance strategies in this group of patients. A positive correlation between dolutegravir- associated weight gain with hypertension has been reported in other larger studies involving both ART naïve and ART exposed PLHIV before and after starting dolutegravir. These studies have had generally older patients with more risk factors for incident hypertension [8,19,20]. One such studies including data from the RESPOND cohort which is a consortium cohort of PLWH with a median age of 43 from 37 countries in Europe and Australia demonstrated that participants receiving INSTIs have a higher incidence of hypertension than those receiving NNRTIs [27]

In our study, we replicated these findings in a relatively younger population (median age; 31 years) with generally minimal other risk factors for hypertension, i.e., at baseline, majority were HIV stage I, were physically active, had normal weight and had normal median creatine levels. With other positive attributes to dolutegravir like having a high genetic barrier to resistance, having less drug- drug interactions, being a once daily drug, good tolerability and low cost of production, DTG is likely to remain part of preferred first line ART for PLHIV. The likely association with hypertension still warrants more research, especially in sub-Saharan Africa where resources for screening as well as treatment for hypertension are still limited. However, this growing evidence to suggest a tendency towards worsening high blood pressure in these patients may warrant intensified blood pressure surveillance in programmatic settings and as well aggressively optimize blood pressure treatment in hypertensive patients on DTG based ART.

Our study had limitations. With our study design, we could not demonstrate if the blood pressure increase we demonstrated is higher than what would be expected in patients on other ART regimens with similar increments in weight given we did not have a comparator group on other ART regimens. Additionally, we also had a limited period of follow-up (48 weeks). Despite the limitations, we had different strengths. Pregnant and breastfeeding mothers who are at risk of gestational hypertension were excluded. We also excluded participants who were on hypertension treatment as well as those with poor adherence to ART as all these could be potential confounders in the hypertension trajectories we report.

## Conclusion

We demonstrated a positive linear relationship between weight gain and mean arterial pressure in Ugandan persons with HIV over 48 weeks on tenofovir disoproxil/ lamivudine/ dolutegravir. We provide additional evidence to suggest that the noticed weight gain after starting dolutegravir based ART may be associated with a heightened risk of incident hypertension.

## Abbreviations

**ART**: Anti-retroviral Therapy
NRTI: Nucleoside Reverse Transcriptase Inhibitors
NNRT: Non- Nucleoside Reverse Transcriptase Inhibitors
DTG: Dolutegravir
WHO: World Health Organization
PLHIV: People Living with HIV
CDC: Center for Diseases Control
BMI: Body Mass Index
GPAQ: Global Physical Activity Questionnaire
AUDIT: Alcohol Use Disorders Identification Test
FBG: Fasting Blood Glucose
2hBG: 2-hour Blood Glucose
MET: Metabolic Equivalent of Task
LDL: Low Density Lipoprotein
HDL: High Density Lipoprotein
